# Interval timing relative to response inhibition in the differential reinforcement of low-rate responding in normally developing young adults

**DOI:** 10.1038/s41598-023-39160-z

**Published:** 2023-07-24

**Authors:** Tsung-Han Yang, Ruey-Ming Liao, Chung-I. Su, Chun-Yi Chien, Chan-Tat Ng, Nai-Shing Yen

**Affiliations:** 1 Department of Psychology, National Chengchi University, No. 64, Sec. 2, Zhih-Nan Rd., Taipei, 116011 Taiwan; 2 Institute of Neuroscience, National Chengchi University, No. 64, Sec. 2, Zhih-Nan Rd., Taipei, 116011 Taiwan; 3 Research Center for Mind, Brain, and Learning, National Chengchi University, No. 64, Sec. 2, Zhih-Nan Rd., Taipei, 116011 Taiwan; 4 Department of Psychology, Asia University, No. 500, Lioufeng Rd., Taichung, 413305 Taiwan; 5 Department of Medical Research, China Medical University Hospital, China Medical University, No. 91, Xueshi Rd., Taichung, 404333 Taiwan

**Keywords:** Psychology, Human behaviour

## Abstract

With recent proposal suggesting the multifaceted nature of impulsivity, researchers have been intrigued by the question of whether the impulsive behaviour measured in the traditionally psychological paradigms is unitary. One such paradigm, the differential reinforcement of low-rate responding (DRL), has been used to assess response inhibition, but its underlying mechanism has still been debated. In present research, we examined and differentiated the effects of both response inhibition and interval timing on a multisession DRL-10 s (DRL-10 s) in a large sample of normally developing young adults, as well as with three other measures including the stop-signal reaction task (SSRT), time production task-10 s (TPT-10 s), and the Barrett impulsivity scale-11 (BIS-11). The results showed that behavioural changes existed in DRL. As the task sessions progressed, there was an increase in both reinforcement probability and peak time, but a decrease in burst responses. Most importantly, both principal component analysis and generalized multilevel modeling yielded consistent results that as the task progressed, there was an increasing involvement of the TPT in the late sessions of DRL. However, none of the effect of SSRT was found. In sum, the differential degrees of involvement of the timing process, relative to response inhibition, were observed in DRL.

## Introduction

In the domains of psychological and psychiatric research, response disinhibition is considered a primary facet of impulsive action. It refers to the inability of an individual to refrain from, withhold, or even cancel a certain action once it is initialized^1–7^, and often seen in children with attention-deficit/hyperactive disorder (ADHD) as well as individuals with severe alcohol, drug, and other substance addiction^8–13^. To assess this inhibitory-related impulsivity, numerous psychological paradigms such as Go/No-Go (GNG), stop-signal reaction task (SSRT), and differential reinforcement of low-rate responding task (DRL) have been conducted by researchers, those accumulated an abundance of evidence that high-impulsive individuals produce more disinhibiting-related or premature-like responses than low-impulsive ones^5–7,14,15^. However, as growing evidence indicates the multifaceted nature of impulsivity, whether the impulsive behaviour assessed in those traditional paradigms is unitary has been challenged; there may be more than one or even multifaceted behavioural components measured in a task. For example, in SSRT and GNG, researchers found that not only the response inhibition, but also the performance (or error) monitoring were involved^16,17^. One such paradigm, the DRL, has also been discussed for the measured behaviours since it was proposed^18^.

DRL has a long history of being used to assess the degree of impulsiveness on both human children and the clinical population, and animals with specific pharmacological injection in the laboratory. In addition, this task is demonstrated useful in reducing certain inappropriate or hazardous behaviours in the applied settings, such as inappropriate or excessive questions asked from behaviourally disturbed children or adults with intellectual disabilities^19,20^, stereotypy from people with mental retardation^21^, and even rapid eating^22^. Both the validity in measuring impulsivity and the practicability for clinical use have made the DRL representative to study impulsivity. In the design of a DRL, a behavioural response would be reinforced only after withholding a specific time interval greater than the criterion time interval between two responses (the inter-response time; IRT). Any response within the criterion time interval led to no reinforcer for the subject. And the “timer”, which governs the time interval, will reset until the following response fulfils the criterion time interval^23^. A modification that can be applied to DRL schedule is the implementation of a limited-hold (LH) contingency, which sets a maximum duration for IRT for being reinforced^24^. Under this operant procedure, DRL has been primarily defined as a measurement of response inhibition owing to the requirement for ‘withholding’ responses until the criterion time elapses. The behavioural indices, such as the efficiency ratio (reinforcement rate), burst responses (the rapid responding within IRT < 2 s), peak rate (the motivation of the subjects), and peak time (the expected IRT for reinforcement), were mostly calculated to investigate the DRL. In a study of emotionally disturbed hyperactive and non-hyperactive children, two groups of children produced a similar number of reinforced responses in a DRL-6 s schedule (DRL-6 s). However, compared with non-hyperactive children, hyperactive children were relatively unable to refrain from emitting a high number of non-reinforced responses, resulting in a lower efficiency ratio performance, that is, a lower reinforcement rate (the number of reinforced responses/total responses). These DRL indices demonstrated an effective discrimination of hyperactive behaviors, and the measured inhibitory-related performances were found to be unrelated to neither age nor IQ in children^13^. Particularly, previous research has found that individuals with high impulsivity tend to produce a very short IRT, rapidly non-reinforced action between two responses (e.g. IRT < 2 s). This type of responding, defined as the burst responses, is usually observed in large quantities as the first peak on a bimodal IRT distribution from the previous DRL results^25–27^. For example, the high-impulsive rats being either selected by the lower efficiency ratio behaviourally or induced by the injection of d-amphetamine or cocaine were found to have a higher number of burst responses than that of the low-impulsive rats on distinctive interval-timing DRL schedules^28–30^. The burst responses hence has been considered a prominent index of heightened impulsivity, and even loss of self-control in the DRL behaviour^29,31–33^.

Despite the aforementioned studies, an alternative explanation has been proposed for the behaviour measured in the DRL tasks. The operant behaviour of withholding a response might involve restraining the motor action, but ‘for how long’ each response should be withheld would be much critical to the subjects in any of the DRL schedules. Given that the criterion time interval is typically set within the range of seconds (e.g., 5, 10, 20, or 36 s) in most DRL, the behaviour of interval timing, which refers to how individuals perceive and estimate the time durations accurately, has been an alternative suggestion in DRL behaviour^26,29,33–37^. This type of response is usually observed as the second peak on the bimodal IRT distributions, and mostly located at the expected time interval for the requirements of a DRL, defined as the peak time^38–41^. A striking piece of evidence was from Wogar et al.^42^, who found that the destruction of 5-hydroxytryptaminergic (5-HTergic) pathways in Wistar rats caused a reduction in both peak time and reinforcement frequency, but did not alter the rates of burst responses and total responses significantly. This finding implied an intimate association between the interval-timing behaviour and reinforcement contingency, and detached the influence of response inhibition from DRL^38^. Another piece of evidence was obtained from a study of the transition of reinforced timing intervals in children. In the DRL-5 s condition, 7 out of 11 children reached a mean proportion of reinforced IRTs greater than 0.30, and the mean IRT was equal to or greater than the criterion time interval of 5 s. After the reinforced criterion was changed to 20 s without additional instruction, 6 out of the same 11 children still attained a similar level of performance in the DRL-20 s. These results again were unrelated to either IQ or receptive language assessment in children, thus supporting the critical role of interval timing, or even a flexibly adjusting behaviour in DRL.

While plentiful evidence supported response inhibition or interval timing, some have suggested that it might be both behaviours, not just one of them, underlying the DRL^26,29,33–36^. For example, in our previous experiment, as the rats’ efficiency ratio increased from the 7th to the 14th day, we found an increasing peak time to the criterion time interval, but also a decreasing burst responses in high-impulsive rats^30^. However, whether both behaviours modulated the DRL simultaneously or alternatively on different processes or by different orders has remained unknown. One of the presumptions was the multiple behavioural processes, which proposed that DRL behaviour may be established from the inhibition of non-reinforced responses, and then gradually transferred to the temporal process^43^, but this processes has not yet been examined in previous DRL researches. Also, it is noted that few studies have conducted DRL on normally developing adults as main research targets, and utilized a large sample design to explore the DRL behaviour. The selected subjects in most DRL researches were the animals with specific pharmacological injections or human/children with specific behavioural tendencies or psychiatric disorders. Choosing normally developing adults as participants can exclude the potential influence from certain psychiatric or pharmacological status to this scheduled-controlled behaviour, and a large sample size will improve the reliability of research results. Therefore, in the present study, our aim was to investigate how normally developing young adults responded, and examine and differentiate the effects of both response inhibition and interval timing on a multisession DRL systematically using a large sample design. In experiment, a DRL-10 s, a stop-signal reaction task (SSRT)^44^, a time production task (TPT)^45^, and the Barrett impulsivity scale-11 (BIS-11)^46^ were conducted. Previously, the poor performance of the stop-signal response in SSRT and the higher score of the motor-impulsive dimension in BIS-11 were considered the behavioural and self-reported disinhibition^1,5,47–51^. The accuracy of TPT is considered an index of interval timing^52,53^. We hypothesized that if response inhibition is involved in DRL behaviour, then the performance of SSRT, BIS-11, and the burst responses would predict the efficiency ratio of DRL. On the contrary, if interval timing is involved in DRL behaviour, then the performance of TPT and the peak time would predict the efficiency ratio of DRL. Alternatively, if both behaviours are involved in DRL, according to the presumption of multiple behavioural processes, we hypothesized the performance of SSRT, BIS-11, and the burst responses would predict the efficiency ratio in the earlier sessions of the multisession DRL task. Relatively, the performance of TPT and the peak time would predict the efficiency ratio in the later sessions of the DRL task.

## Results

### Inter-response time (IRT) and DRL indices

**Figure 1:**
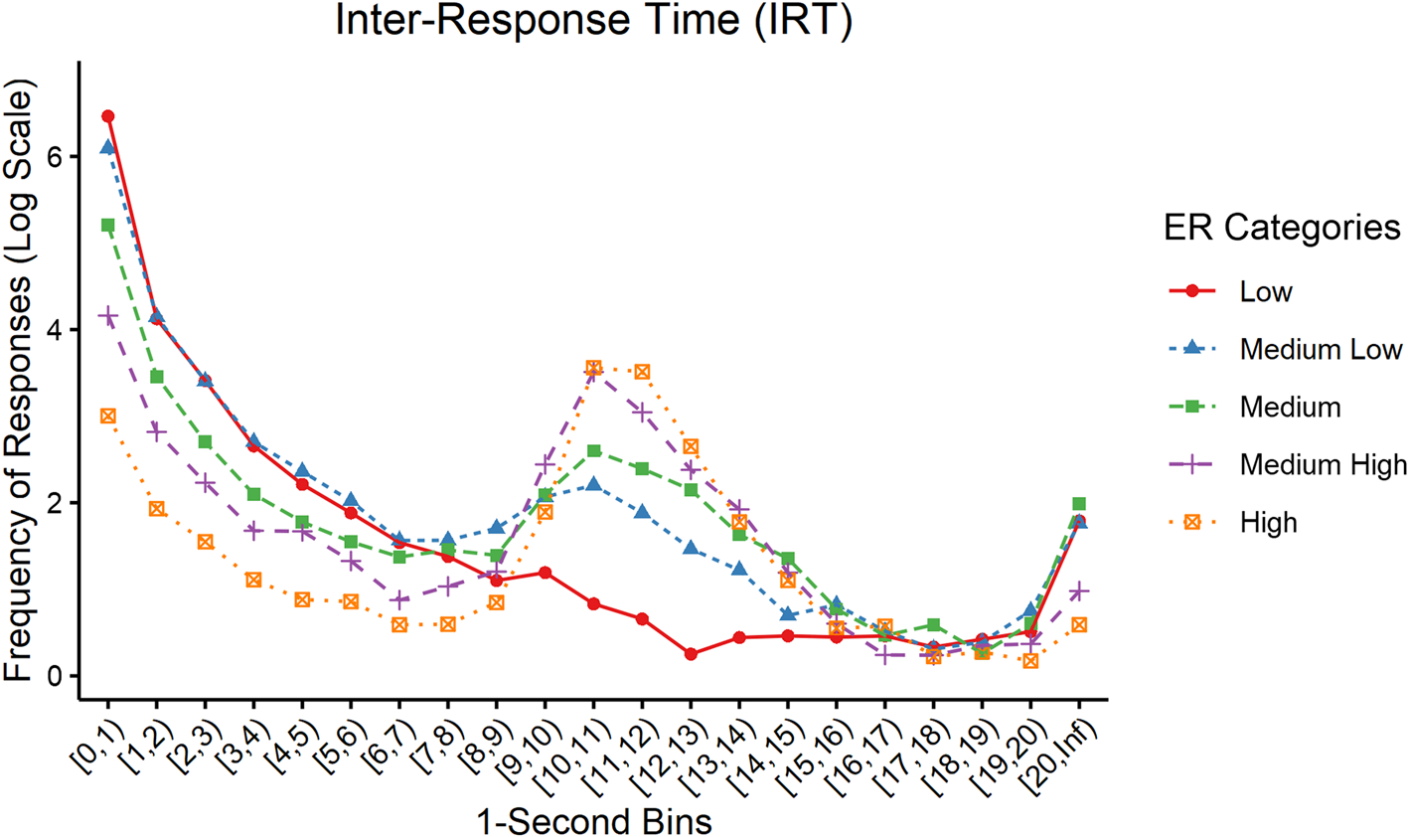
Overall distributions of inter-response times (IRT) stratified by efficiency ratio (ER) categories across all Differential Reinforcement of Low-rate responding - 10s (DRL-10) sessions. Each point represents the average log-transformed response frequency for a particular IRT bin within a specific ER category. Lines connect the points within each category to illustrate the trends in response frequency across IRT. The IRT values (1-sec bins) are plotted along the x-axis, and the log-transformed response frequencies are plotted along the y-axis.

The individual differences were examined in the IRT distribution and behavioural indices of DRL in a final sample of 152 participants (mean age = 21.73, *SD* = 2.18; 112 females and 40 males), after excluding 15 participants with no reinforced response in any of the DRL sessions. Figure 1 compared the distinct levels of ER that were formulated based on the quintile distribution of average logit-transformed ER across all DRL sessions, comprising the low, medium–low, medium, medium–high, and high ER groups. Overall, the IRT responses showed that participants with greater ER had fewer non-reinforced responses, particularly in the time range < 2 s (burst responses), but more reinforced responses in the time range within 10–13.99 s (see Fig. 1).

**Figure 2:**
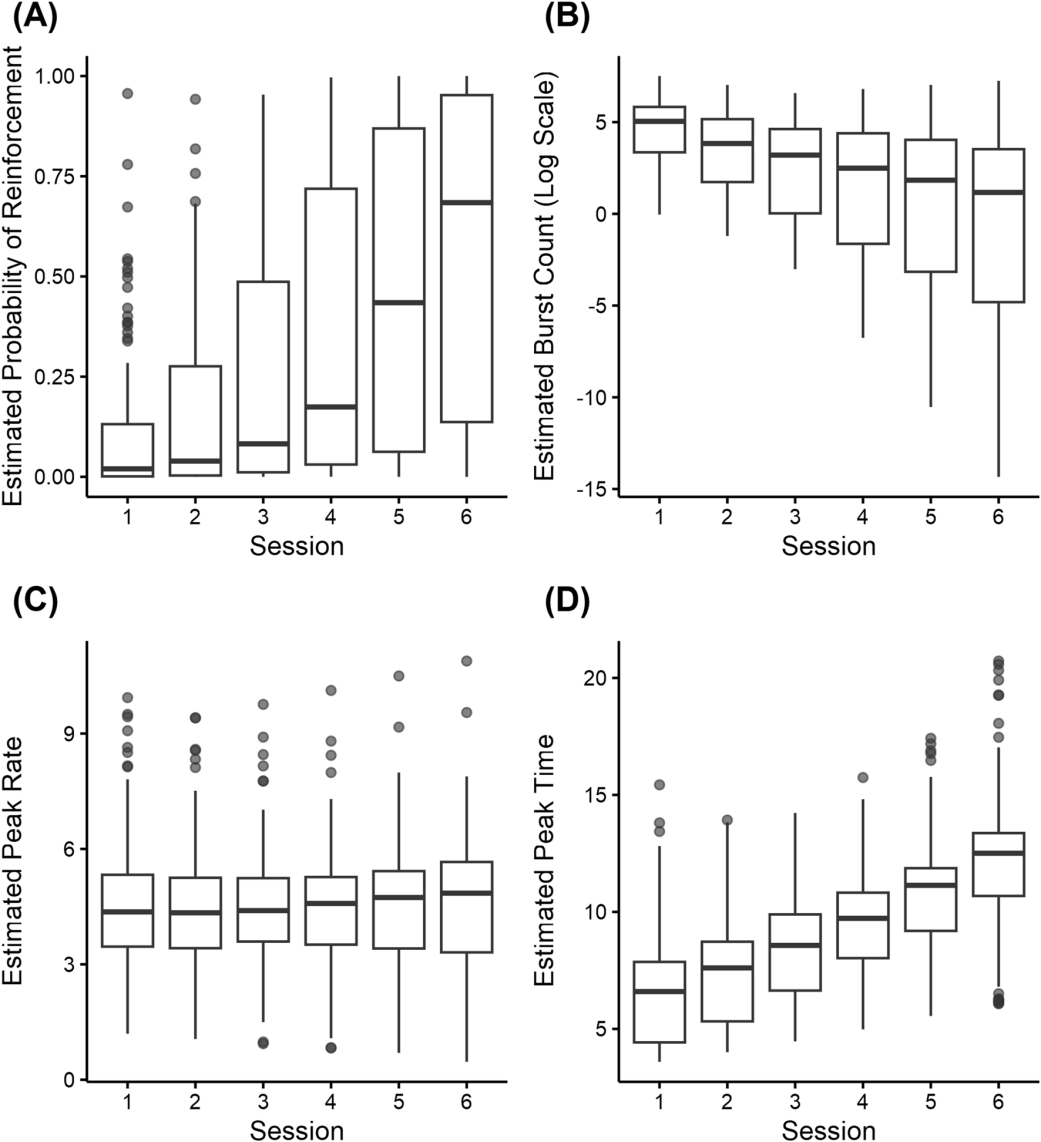
Mixed effect model predictions of reinforcement probability, burst responses, peak rate, and peak time across all sessions. These panels present the distribution of estimated parameters derived from the mixed-effects models for each dependent variable, considering random effects: (**A**) the probability of reinforcement, (**B**) the burst responses (log scale), (**C**) the peak rate, and (**D**) the peak time. For each panel, the y-axis shows the respective estimated parameters, and the x-axis denotes the task session. Boxplots represent the distribution of these estimated parameters across participants in each session. The models used to generate these predictions were specified with a binomial distribution for reinforcement, a Poisson distribution for burst responses, and Gamma distributions for both peak rate and peak time. Each boxplot encapsulates the interquartile range, median, and outliers of the estimated. Across sessions, the (**A**) probability of reinforcement and (**D**) peak time generally increased, while the (**B**) burst responses tended to decrease. The (**C**) peak rate, however, did not demonstrate a clear pattern of change across sessions.

To further investigate the behavioural changes during a multi-session DRL, a generalised multilevel modelling (GMLM) approach was used to estimate task session parameters for the four DRL indices—reinforcement (reinforced or not; whether the IRT of a response fell within 10–13.99 s), burst responses (number of responses with an IRT < 2 s in each session), peak rate (maximum number of non-burst responses calculated by averaging 4 consecutive 1-s time bins in each session), and peak time (the estimated time derived from the peak rate in each session)—while accounting for variability in the intercepts and slopes for session across individuals (see Fig. 2).

A GMLM with a binomial distribution applied for the dichotomous reinforcement data revealed a significant effect of task session (estimate = 1.031, *Z* = 13.30, *p* < 0.001), indicating an increase in reinforcement probability with task sessions. Further, given the presence of excessive zero burst counts, we fitted the burst responses data with a zero-inflated Poisson model to account for excessive zeros. A negative effect of task session on burst responses (estimate = − 0.961, *Z* = − 10.93, *p* < 0.001) was found, indicating a decrease in the number of burst responses with task sessions. Finally, we fitted peak rate and peak time, respectively, using a GMLM with a gamma distribution for dealing with continuous positive outcomes with non-normal distribution. The model showed no change in peak rate across all task sessions (session estimate: − 0.011, *Z* = − 0.634, *p* = 0.526), implying that the peak rate remained stable over the course of the DRL sessions. However, a positive relationship was observed between peak time and task session (estimate: 0.130, *Z* = 9.274, *p* < 0.001), indicating an increase in peak time as task sessions progressed. In sum, the results indicated that as the task sessions progressed, both reinforcement probability and peak time increased, burst responses decreased, while peak rate remained unchanged (see Fig. 2).

### Principal component analysis (PCA) to test DRL behaviour

In order to examine our research hypotheses, a PCA was conducted. The results of PCA yielded eight new components with eigenvalues ≥ 1, together accounting for 75.063% of the variance of all items. The first to sixth components were linear composites of the DRL indices and BIS-11, the seventh and eighth were the composites of TPT and SSRT respectively. The findings are described: (1) the first component reflected the motivation of participants to perform the DRL. Loadings were mainly positive for both peak rate in session 2–6 and efficiency ratio in session 3–6, also burst responses in session 5–6 and peak time in session 6. It suggested that the higher the motivation, the greater the efficiency ratio in DRL. (2) The second component contained the operant behaviours in the middle sessions of DRL. Loadings were positive for both efficiency ratio in session 2–4 and peak time in sessions 2–5, and negative for burst responses in sessions 2–4. (3) The third component was the composites of all burst responses except that in session 1. Although we expected both burst responses of DRL and false rate of SSRT might reflect similar response inhibition, the results that loadings were significant for burst responses without SSRT implied another mechanism underlying the burst responses. (4) The fourth component was the composites of all measurements within BIS-11, which implied the measured construct in self-reported impulsivity might differ from that in behavioural impulsivity. (5) The fifth component contained the operant behaviours in the initial sessions of DRL. Loadings were positive for both efficiency ratio and peak time in session 1–2, negative for burst responses in session 1–2, and positive for peak rate in session 2. (6) The sixth component reflected the relationship between peak rate and burst responses. Loadings were positive for peak rate in session 1–4 and burst responses in sessions 1–2, suggesting the higher the motivation to perform DRL, the lower the number of burst responses were made.

Most importantly, the seventh and eighth components answered the research question. (7) The seventh component contained the timing-related behaviours in the late sessions of DRL with TPT. Loadings were positive for both efficiency ratio and peak time in session 5–6 and the accuracy of TPT, which suggested that while participants’ efficiency ratio reached the highest in the late sessions of DRL, the timing estimation behaviour was involved. On the contrary, (8) the eighth component only contained the behaviour of SSRT, not with any burst responses and efficiency ratio as predicted, even with another measurement of DRL, BIS-11, and TPT. Hence the hypothesis of response inhibition was not supported. These extracted principal components not only revealed the absence of response inhibition influence, but highlighted the significant changes in behavioral involvements across task sessions: a progressive increase in timing-related behavior throughout the DRL (see Table 1).

**Table 1:** Principal component analysis (PCA) yielding 8 rotated components (*N* = 152).

	Rotated components							
	1	2	3	4	5	6	7	8
Eigenvalues	**9.134**	**3.368**	**2.762**	**2.290**	**1.648**	**1.189**	**1.095**	**1.033**
variance	**30.447%**	**11.227%**	**9.205%**	**7.634%**	**5.494%**	**3.962%**	**3.650%**	**3.443%**
DRL_S1 ER(logit)	.124	.285	− .050	.056	**.796**	.200	.048	.000
DRL_S2 ER(logit)	.238	**.576**	− .117	.084	**.592**	.164	− .018	− .032
DRL_S3 ER(logit)	**.382**	**.710**	− .174	.138	.244	.158	.118	− .065
DRL_S4 ER(logit)	**.591**	**.540**	− .226	.057	.110	.138	.188	− .107
DRL_S5 ER(logit)	**.668**	.264	− .257	.078	.105	− .037	**.379**	.032
DRL_S6 ER(logit)	**.731**	.169	− .206	.043	.222	− .082	**.349**	− .038
DRL_S1 BR	.136	− .162	.296	− .067	**-.548**	**-.551**	− .182	.068
DRL_S2 BR	.059	**-.325**	**.487**	− .104	**-.523**	**-.352**	− .019	.109
DRL_S3 BR	− .014	**-.476**	**.694**	.048	− .210	− .270	.058	.105
DRL_S4 BR	− .045	**-.344**	**.801**	.007	− .062	− .240	.028	.061
DRL_S5 BR	**-.364**	− .093	**.792**	.013	− .093	.094	− .155	− .050
DRL_S6 BR	**-.366**	.044	**.798**	.052	− .101	.138	− .053	− .103
DRL_S1 PR	.165	− .039	.023	− .043	.073	**.811**	.083	− .004
DRL_S2 PR	**.349**	.040	− .028	− .091	**.449**	**.452**	− .115	.229
DRL_S3 PR	**.519**	.267	− .123	− .049	.079	**.518**	− .168	− .156
DRL_S4 PR	**.677**	.290	− .129	− .002	− .077	**.466**	− .034	− .182
DRL_S5 PR	**.819**	.046	− .145	.080	− .002	.236	.030	− .042
DRL_S6 PR	**.891**	− .031	− .037	− .026	.063	− .002	.014	.088
DRL_S1 PT	.058	.089	− .066	.155	**.759**	− .058	.093	− .045
DRL_S2 PT	.063	**.488**	− .248	.087	**.544**	− .155	− .015	− .166
DRL_S3 PT	.004	**.784**	− .076	.097	.275	.042	.018	− .019
DRL_S4 PT	.177	**.683**	− .255	− .013	.105	− .055	.280	.198
DRL_S5 PT	.214	**.430**	− .142	.005	.089	− .157	**.639**	.168
DRL_S6 PT	**.301**	.171	− .383	− .005	.140	− .143	**.566**	.107
SSRT_false rate	− .068	.001	− .011	.089	− .101	− .039	− .014	**.921**
TPT_accuracy	.031	− .043	.134	− .077	− .011	.274	**.655**	− .170
BIS_total	.029	.057	.014	**.992**	.072	− .018	− .020	.025
BIS_attentional	.008	.085	.024	**.752**	.176	.004	.189	.110
BIS_motor	.051	.002	− .016	**.828**	.099	− .040	− .138	.004
BIS_non-planning	.008	.056	.026	**.874**	− .050	− .005	− .059	− .033

ER(logit) = efficiency ratio (with logit transform); BR = burst responses; PR = peak rate; PT = peak time; SSRT = stop-signal reaction task; TPT = time production task; BIS = Barrett impulsivity scale.

Loadings ≥ 0.3 are in bold.

### Generalized multilevel modelling (GMLM) to predict reinforcement in DRL

Concerning the potential hierarchical structure and non-normal distributions in our data, we complemented the analysis with a GMLM that included individual variation in the DRL indices, SSRT, TPT, and BIS-11 to fit the reinforcement data and test our research hypotheses. An attempt was made to construct a comprehensive model incorporating all sessions and interaction terms, capturing the joint influence of all measurements. But the convergence issues arising from the complexity of such a model made this approach unfeasible. Consequently, we analysed each DRL session separately: each model was structured with all variables as fixed effects, along with a random effect in the intercepts and slopes for session account for inter-individual variability; these models were constructed using a binomial distribution with a logit link function, applied separately to each session to track the temporal changes in these relationships.

Table 2 shows the estimates of each variable for each session. The DRL indices, comprising the number of burst responses, peak rate, and peak time, consistently displayed the significant effects in predicting reinforcement in all sessions (all *p*s ≦ 0.032, except for peak rate in Sessions 2 and 3). Similar to the findings of PCA, the accuracy of TPT significantly predicted the reinforcement probability in the last and even middle sessions of DRL (Session 3: *p* = 0.052; Session 5: *p* = 0.040), but the false rate of SSRT did not reveal the effect on the reinforcement in any of the sessions (see Table 2). On the other hand, the total BIS-11 scores were significantly linked to DRL reinforcement in sessions 3 and 4 (both *p*s ≦ 0.030). Subsequent analysis using the three sub-dimension scores of the BIS-11 instead of the total scores revealed that only attentional impulsivity could predict DRL reinforcement (sessions 3–6: all *p*s ≦ 0.011). It should be noted, however, that such a relationship was positive, with higher attentional impulsivity being associated with a greater likelihood of DRL reinforcement.

**Table 2:** Generalized multilevel regressions for Reinforcement by DRL indices (burst responses, peak rate, peak time), SSRT, TPT, and BIS-11 (*N* = 152).

Variable	DRL Session1		DRL Session2		DRL Session3		DRL Session4		DRL Session5		DRL Session6	
	Est	*p*	Est	*p*	Est	*p*	Est	*p*	Est	*p*	Est	*p*
Intercept	**−7.569**	** < .001**	**− 7.964**	** < .001**	**− 7.254**	** < .001**	**− 6.841**	** < .001**	**− 13.869**	** < .001**	**− 8.870**	** < .001**
Burst responses	**− 4.911**	** < .001**	**− 5.530**	** < .001**	**− 4.813**	** < .001**	**− 4.498**	** < .001**	**− 11.912**	** < .001**	**− 6.810**	** < .001**
Peak rate	**− 0.567**	** < .001**	0.350	.164	0.504	.060	**1.393**	** < .001**	**0.821**	** < .001**	**1.757**	** < .001**
Peak time	**0.402**	**.011**	**0.607**	**.005**	**0.813**	** < .001**	**0.946**	** < .001**	**0.429**	**.032**	**0.957**	** < .001**
SSRT_false rate	− 0.083	.505	− 0.095	.602	− 0.142	.490	− 0.270	.175	− 0.032	.843	− 0.287	.072
TPT_accuracy	0.073	.556	0.256	.163	0.415	.052	0.228	.278	**0.343**	**.040**	0.263	.103
BIS-11_Total	− 0.026	.823	0.230	.180	**0.521**	**.008**	**0.407**	**.030**	0.277	.073	0.294	.051
Random intercept variance	1.356	−	3.848	−	5.382	−	5.125	−	3.426	−	3.296	−

Significant values are in bold.

## Discussion

In psychological and psychiatric research, selecting an appropriate and valid task paradigm to measure specific psychological or psychiatric constructs is crucial for researchers. Traditionally, paradigms such as GNG, SSRT, and DRL have been used to assess response inhibition predominantly. However, with the recent proposal of the multifaceted nature of impulsivity, researchers have been intrigued by the question of whether the impulsive behaviour measured in those tasks is unitary. In the present study, in addition to investigating the DRL behavioural acquisition, we examined the effects of both response inhibition and interval timing on a multisession DRL systematically. The main results were as follows: (1) behavioural changes in human participants existed in acquiring a DRL task; (2) differential degrees of involvement of the timing process relative to response inhibition were observed in the present test of DRL behaviour.

In the recent studies of impulsivity, a wide range of behaviours has been revealed through different action-related measurements, such as motoric inhibition, waiting, and even temporal regulation. On the neurometabolic evidence, while previous studies have proposed the critical structural and functional role of the striatum, including both dorsal striatum (DS) and nucleus accumbens (NAc), in impulsivity, researchers still discovered distinctive findings associated with specific neurochemicals in different neural substrates for various forms of impulsive action^5^. For example, excitotoxic lesions of the dorsomedial striatum, but not the NAc core, impair the performance of stop-signal responses in Lister-hooded rats^54,55^. Relatively, the depletion of dopamine (DA) within the NAc by 6-hydroxydopamine (6-OHDA) lesions alters premature responses in Lister-hooded rats in 5-choice serial reaction time task (5-CSRTT)^56^. This behavioural and neural evidence together elucidated the complex nature of impulsivity^5,6,26,33,57^. As one of the representative paradigms, DRL has still been debated on the underlying mechanism, such as response inhibition^58,59^, interval timing^41,60–62^, or both^26,29,35^. Our human results showed that as the task sessions progressed, there was an increase in both reinforcement probability and peak time, a decrease in burst responses, while peak rate remained unchanged. Moreover, to examine our two alternative hypotheses, both PCA and GMLM were conducted. The results of both tests consistently revealed that as the task sessions progressed, there was a significant effect from the accuracy of TPT in the late sessions of DRL, which implies that when participants had the highest performance in the late sessions of DRL, what they had performed might have estimated or calculated the timing interval precisely. In contrast, none of the DRL indices, particularly the efficiency ratio and burst responses, had significant loadings with both the false rates of SSRT and the self-reported motor impulsivity in BIS-11 by PCA; and none of the effects of SSRT and self-reported motor impulsivity predicted the reinforcement in DRL by GMLM. These results imply that when participants performed the DRL, the behaviour of response inhibition may not be involved. Therefore, the hypothesis of interval timing, rather than response inhibition, was supported in this study.

It is noted that this result was partially consistent with the multiple behavioural processes presumption that DRL behaviour would be transferred to the temporal-related process^43^. However, according to the same presumption, performance in the early sessions of DRL would be expected to relate to the responses disinhibition of SSRT or motor impulsivity of BIS-11, but neither was revealed in the present study. A possible reason for the distinctive findings regarding response inhibition between the previous and present studies might be the differences in subject selection. As mentioned, most DRL experiments have been applied to subjects with impulsive or impulsive-like traits, such as children with hyperactivity^13^, children, adolescents, or adults with ADHD related history or other mental retardation^12,63,64^, rats with spontaneous hypertension^26^ or even being administered with specific psychomotor stimulants such as amphetamine or cocaine^43^. These impulsive or impulsive-like subjects, dominated by their behavioural tendency or being manipulated to disinhibit their initial or prepotent actions, tend to generate a high frequency of rapidly motoric responses in response to certain stimuli. This may be the cause of the non-reinforced responses, or even the burst responses in previous DRL subjects^13,29–33^. For example, the alcohol-dependent patients (ADP) with a cluster-B personality disorder (PD) were found to have the impaired performances on both SSRT and DRL-6 s^65^. The Sprague–Dawley rats being injected the cocaine treatment had a higher number of total responses and burst responses than the vehicle treatment condition in the DRL-12 s^28^. In current study, none of the relationship was found between the burst responses and the other measurement of response inhibition as expected. This led to an interesting question of whether burst responses were not the index of response disinhibition, then what other behaviour might they reflect in normally developing young adults? One possibility we speculated here was the collateral behaviour^25,66^. During the DRL experiment, subjects might generate this type of pressing behaviour to test the possibility between responses and reinforcements, and try to control the subsequent behaviour. Even though this large quantity of responses was not always reinforced, it still helped the subjects consider the possible relationship between their responses and outcomes. For example, in the study by Gaucher et al. (2015), when the 2.6 to 7 years old participants were shifted from the DRL-5 s to the DRL-20 s condition, the number of burst responses increased immediately in the first DRL-20 s session for all participants, indicating that transferring the DRL-reinforced rules led children to change their behavioural patterns quickly^25^. In our study, some of the participants expressed their suspicion that the reinforcement rule might be based on either rapid pressing or a consecutive sequence of a large number of pressing responses, both resulted in producing a high number of burst responses in experiment.

Our results providing support for the interval timing hypothesis are conceivable. In the DRL, participants were only reinforced if their responses fell within the IRT of 10–14 s. In order to receive reinforcements, what participants had to was to estimate and calculate each 10 s accurately. This is similar to the behaviour performed in the TPT-10 as well. It’s interesting that some arguments propose the short IRT responses, such as burst responses in DRL and stopping responses in SSRT, are also a form of interval timing behavior. However, according to Buhusi and Meck (2005), the short-duration intervals-timing, particularly in the millisecond range, is primarily controlled by the motor timing system. This system plays a significant role in motor control, speech generation, recognition, and other related behaviors. On the other hand, intervals-timing ranging from seconds to minutes is primarily controlled by the interval timing system, which has a critical impact on time estimation and decision-making and so on^67^. These observations elucidate why the DRL researchers regarded the longer-IRT counting as an interval-timing or even a temporal control task^41,60–62^. As mentioned earlier, DRL has been documented in children even at a very early age^25,60,68,69^. Pouthas (1981) found that children aged 8–24 months could reach an efficiency ratio between 0.2 and 0.3 in DRL-5 s^68^. Weisberg and Tragakis (1967) found that children aged 15 to 41 months reached the efficiency ratio, even superior to 0.4 in both DRL-10 s and -18 s^60^. In addition, although the current study was a simple behavioural research without measuring neural responses, in our previous rat study, through the scanning of in vivo proton magnetic resonance spectroscopy (^1^H-MRS), a significant group difference was revealed in the NAc that high impulsive rats had lower glutamate (Glu) concentration than low impulsive rats, whereas no such difference was revealed on the Glu and the other measured metabolites in DS^30^. This result was similar to the finding of neural profile differences segregated by using 5-CSRTT in Caprioli et al. (2014), where the high-impulsive rats screened by the worse performance in 5-CSRTT had a decreased concentration of glutamic acid decarboxylase in the NAc core compared with the low-impulsive rats. Since what 5-CSRTT has measured is the ability to wait, namely the ‘waiting’ impulsivity^5,6,15,57^, the results in both tasks might reflect the similar behaviour like waiting or interval-timing responses^5,6,15,30,57,70^. Evidence from aforementioned studies supports the timing-processing nature of DRL behaviour.

In summary, to our knowledge, the current study is the first to examine and differentiate the effects of both response inhibition and interval timing systematically on a multisession DRL in a large sample of normally developing young adults by principal component analysis and generalized multilevel modelling. Our results suggest that as the task sessions progressed, the interval-timing behaviour might have been involved in and contributed to DRL performance. Limitations of the present study and some directions for the future research are described. First, some may suggest that there are different types of motoric responses evaluated by burst responses of DRL and false-rates of SSRT. DRL assesses the burst responses at any time, but SSRT measures the false responses once the stop-signal has begun. This fundamental difference might influence the current results. However, according to our literature reviews, both tasks effectively captured the inhibitory-control nature regardless of this measuring difference^28,65^. Whether the design of both tasks that measure the distinctive response inhibition or not requires more investigation in the future. Second, an experiment without specifically operational instructions is prone to violate human participants' intuition, since adults’ behaviour is rule-governed^71^. Under such circumstances, most participants needed to invest lots of time exploring various possibilities to achieve reinforcements in the DRL. Our findings demonstrated that as the task sessions progressed, participants had learned or inferred that interval timing for at least 10 s was the reinforced approach. This also implies that the cognitive process of learning or inference may be influential to DRL. In the study of Avila et al. (2004), the performance of school-aged boys in the Wisconsin card sorting test and their efficiency in the DRL-10 s task were both associated with the same factor "resistance to interference", which highlights the significance of this construct in the DRL^12^. Moreover, comparing the tasks with and without the explicit instructions to count may have distinctive mechanisms. In the TPT, the behaviour of counting 10 s might primarily operate in the verbally-mediated working memory. However, performing the DRL might be much complicated. Participants might utilize various resources to try, learn, and time the intervals throughout the DRL. Future research could take the relationship between the components of DRL and working memory into account. Finally, the investigation of both brain and neural mechanisms for DRL is still scarce; using a neuroimaging approach may provide more evidence for researchers to decipher the behavioural processes of DRL.

## Methods

### Participants

A total of 171 student participants (mean age = 21.72, *SD* = 2.17; 128 females and 43 males) were recruited from the National Chengchi University, Taipei, Taiwan. All of them were self-reported native Chinese speakers, right-handed, with normal or corrected-to-normal vision, not heavy drinkers or taking any medication, and without any history of neurological or psychiatric disorders. Written informed consent was obtained from each participant before the experiment, and NT$150 was reimbursed after the experiments. All methods were performed in accordance with the ethics principles of Declaration of Helsinki, and the study was approved by the Research Ethics Committee of National Chengchi University.

### Experimental procedures and materials

Upon arrival at the laboratory, each participant was introduced to the aim of this study in a sound-attenuated room and signed an informed consent form if they agreed to participate. They were told to complete three behavioural tasks conducted using a computer and a handwritten self-report questionnaire. The designs of these measurements, the DRL, SSRT, TPT, and the Chinese version of BIS-11 (Li & Chen, 2007) are described below.

#### Differential reinforcement of low-rate responding task-10 s (DRL-10 s)

**Figure 3:**
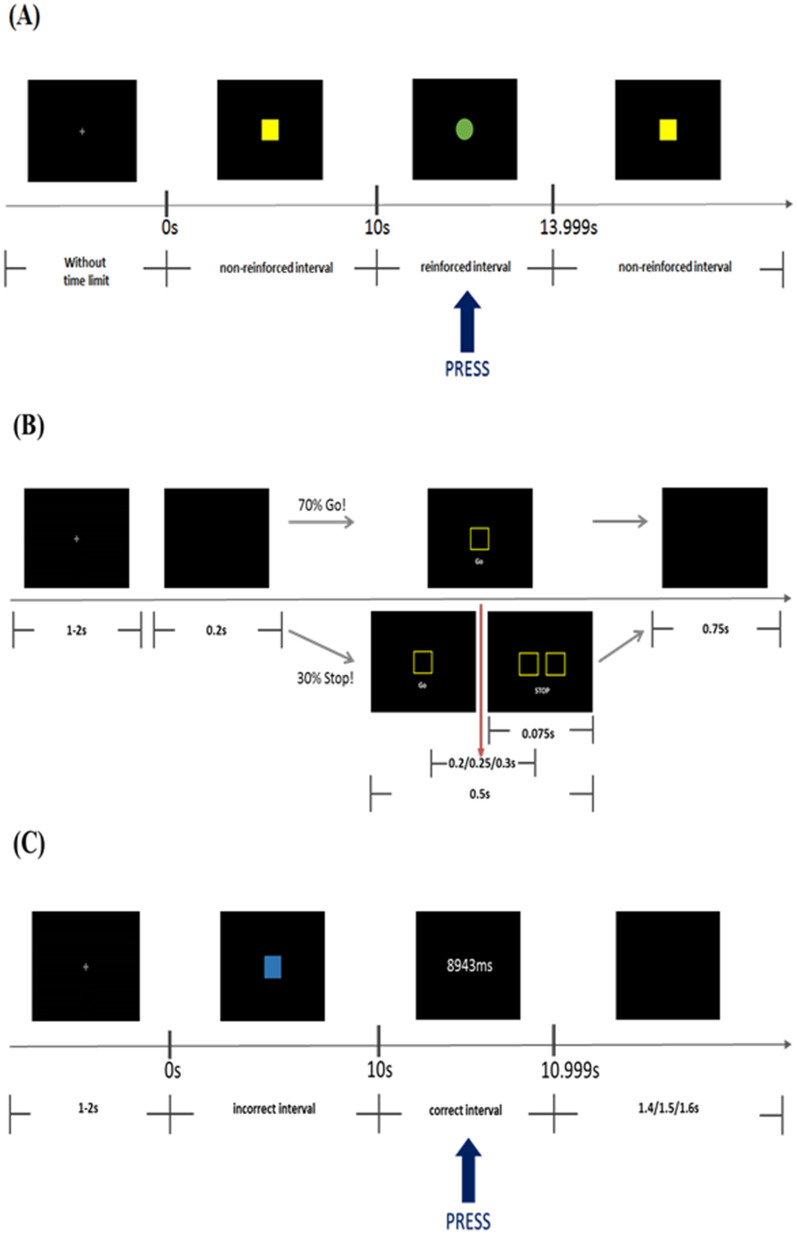
The experimental procedures for the three behavioral tasks. (**A**) Differential reinforcement of low-rate responding task-10 s (DRL-10), (**B**) Stop-signal reaction task (SSRT), and (**C**) Time production task-10 s (TPT-10).

In the DRL-10 s, participants were instructed that their goal was to try and earn as many green circles as possible by pressing a space button, but were not given how to do so exactly from the instruction. This design provided a better ecological analogue to previous animal DRL studies and excluded the influence of humans’ prior knowledge or experience^72^. The more the green circles were shown on the computer screen, the higher the opportunities for participants to receive accumulated rewards. In the task, there were six sessions, each lasting 5 min. At the beginning of each session, a white fixation cross “+” was shown at the centre of the screen without a time limit, and the participant could click the left mouse button when he/she was ready to start the experiment. After clicking, a yellow square was displayed at the centre of the screen as the signal to start and maintained for 5 min, during which each participant could try multiple ways of pressing the space button on a keyboard. A green circle would be shown at the centre of the screen for 1 s as a reinforcement if participants pressed the button within the time interval of 10–13.99 s (10 s with a limited hold of 3.99 s) since the previous response occurred. Conversely, none of the reinforcements would be shown if their response was beside the reinforced time interval (IRT < 10 s and > 14 s), and the yellow square would continue to be displayed until the participants learned how to earn the reinforcement by pressing the space button. Before performing the task, each participant underwent a continuous reinforcement schedule [fixed-ratio (FR) 1] with six trials as practices to learn the association between pressing a space button to an orange square and receiving a green circle on the screen as reinforcement. The total duration of performing DRL-10 s was 30–40 min (see Fig. 3A).

#### Stop-signal reaction task (SSRT)

In the SSRT, participants were instructed that their goal was to press the space button when the go signal appeared but withhold their response when the stop signal appeared as fast and accurately as possible. There were three blocks, each consisting of 50 trials. At the beginning of each trial, a white fixation cross “+” was shown at the centre of the screen for 1–2 s, followed by a short black screen for 200 ms. Then, a square sign was displayed at the centre of the screen for 500 ms as the go signal, to which participants had to press the space button as soon as possible. However, after displaying the go signal, there was a 30% probability to appear the two squares side by side for only 75 ms as the stop signal, and the duration between displaying the go and stop signals was only 200, 250, or 300 ms randomly. Under this condition, participants had to withhold their already-initialised pressing responses as quickly as possible. The duration of each trial was 1250 ms regardless of whether each executing response was correct, and the inter-trial interval (ITI) varied from 1500 to 3500 ms. Before performing the task, there were six trials, including four go-signals and two stop-signal trials, as practice for participants to familiarise themselves with the task. The total duration of the SSRT was 10–15 min (see Fig. 3B).

#### Time production task-10 s (TPT-10 s)

In the TPT-10 s, participants were instructed that their goal was to produce a particular duration (10 s) by pressing a space button in each trial as precisely as possible. There was only one block with 30 trials for this task. At the beginning of each trial, a white fixation cross “+” was shown at the centre of the screen for 1–2 s; then, it was replaced by a blue square sign as the start signal. During each trial, the participants had to count to 10 s themselves and press the space button as a response. Compared to the design of DRL-10 s, participants instructed with TPT-10 s would clearly discern that the correct response was defined by producing a pressing response within 10 to 10.99 s since the square sign had been displayed. After responding, the precise response time would be shown at the centre of the screen for 2 s regardless of whether it was correct (e.g., 10,766 or 8943 ms). This allowed each participant to adjust his/her timing production based on the provided feedback. The inter-trial interval (ITI) was designed as a black screen of 1400, 1500, or 1600 ms presented randomly. Before performing, six practice trials required participants to count 7 s (TPT-7 s) and press the space button to familiarise them with the task procedure. The total duration of the TPT-10 s was 8–10 min (see Fig. 3C).

#### Chinese version of Barrett impulsive scale, version 11 (BIS-11)

BIS-11 is a 30-item self-report questionnaire designed to assess the attentional (the inability to focus or concentrate), motor (the tendency to act without thinking), and non-planning (the tendency to plan without futuring or foresight) impulsivity^73,74^, which has already been translated to various languages with good internal consistency and test–retest reliability^75–78^. The Chinese version of the BIS-11, translated by Li and Chen^46^, used a four-point scale (1 = rarely/never; 2 = occasionally; 3 = often; 4 = almost always/always) to measure, so the total score ranged from 30 to 120. Participants who score higher are considered with higher impulsivity^46,73,74,79^. The internal consistency coefficient (Cronbach’s **α**) for all items was 0.78 in the study by Wang and Yu^80^. The total duration of answering the Chinese version of the BIS-11 was 3–5 min.

The order of the three tasks was randomly presented to each participant, with the DRL-10 s always being administered prior to the TPT-10 s, to prevent participants from inferring that the reinforced responses in the DRL might be related to timing behaviour. After completing all tasks, participants answered the BIS-11 and received a participation fee of NT$150 after the experimenter provided debriefing. Three of the participants did not complete the entire experiment due to their personal arrangements, and one participant’s data was missing due to a recording error on the computer. Data from the remaining 167 participants were analysed. The entire experiment lasted 60–70 min.

### Data collection

The DRL behaviour data were based on the pressing responses recorded by the IRT, and six indices were calculated: (1) the number of total responses, (2) the number of reinforced responses, (3) the number of non-reinforced responses, (4) the number of burst responses, (5) the peak rate, and (6) the peak time. The first three indices were frequency measures, which accumulated the number of total pressing responses, the number of pressing responses within IRT10-13.99 s, and the number of pressing responses within both IRT < 10 s and > 14 s during DRL. The burst response was the summed number of pressing responses within IRT < 2 s, reflecting response disinhibition. The peak rate and peak time were calculated from IRT > 2 s, where a moving average based on four consecutive 1-s bins with 1-s step size was applied to smooth the distribution. The peak rate was calculated as the summed number of pressing responses in the four bins divided by four, which rendered a unit of responses per second for the peak rate, reflecting an individual’s motivation to perform the DRL task. After the maximum of the summed pressing responses of a four-second epoch was identified, the peak time was the average value in ms of all IRTs that fell within those four bins (i.e. the maximal epoch), which showed the time point where participants pressed the button with the highest numbers, reflecting their expected criterion time in DRL^30,81^. In SSRT, the false-responding rate from only stop-signal trials, namely the percentage of false-pressing reactions, was calculated as the index of response disinhibition. In TPT, the response accuracy, that is, the percentage of pressing responses within the time range of 10–10.99 s was calculated as the index of interval timing.

### Statistical analysis

We focused on analysing DRL-related behaviours based on four key indices: reinforcement, burst responses, peak rate, and peak time. Reinforcement, a fundamental aspect of DRL, was treated as a dichotomous outcome variable at the level of individual responses. A response was classified as reinforced if its IRT fell within the range of 10.00 to 13.99 s.

At the session level, burst count was calculated as the total number of burst responses, which were defined as responses with IRT less than 2 s. This provided an insight into the frequency of rapid and impulsive responding during each session. Peak rate and peak time were analysed to further explore the temporal characteristics of DRL at the session level. Peak rate referred to the maximum number of non-burst responses, calculated by averaging four consecutive 1-s time bins within each session. Peak time, derived from peak rate, represented the estimated time at which the peak rate of non-burst responses occurred within each session.

These four indices were analysed using GMLM with appropriate distributions, examining the effects of task session while controlling for random effects in the intercepts and slopes for session across individual participants. Specifically, for the reinforcement index, a binomial distribution with a logit link function was used. For burst responses, a zero-inflated Poisson model with a log link function was used to account for excessive zero counts. For peak rate and peak time, a gamma distribution with a log link function was used. These analyses were performed using R (version 4.2.3) with the *lme4* (1.1–33) and *glmmTMB* (1.1.7) packages.

Second, we tested the hypothesis that the underlying behaviour of DRL was response inhibition or interval timing. Principal component analysis (PCA), which is able to reduce multicollinearity, extract important features from the original data structure, and map the correlated features onto principal components, was performed on the four indices of DRL (efficiency ratio, burst responses, peak rate, and peak time), the false rate of SSRT, the accuracy of TPT, and the scores of the BIS-11 (including the total impulsive score and the three subscale scores). The outcomes of the yielding components with eigenvalues ≥ 1 and loadings ≥ 0.3 were considered critical for interpretation. It should be noted that the efficiency ratio, being expressed as percentage data, resulted in residuals that were not normally distributed. A logit transformation of the data for PCA was required. Also, the distribution of the original peak time was bimodal, hence this measure was transformed by coding the value ≧ 8.2584 (the mean of the original peak time distribution) as 1 and < 8.2584 as 0, making the data as continuous variables for analysis.

Furthermore, generalised multilevel models (GMLMs) were used to predict the reinforced behaviour in DRL. These models extended conventional linear regression modelling by allowing for the clustering of observations within higher-level units and incorporating residuals at both the individual and group levels, was used to predict the reinforced behaviour in DRL as well. For each session, a GMLM using a binomial distribution with a logit link function was fitted. The fixed effects included the standardised values of burst responses, peak rate, peak time, SSRT, TPT, and BIS-11 total score (or the three BIS-11 sub-dimension scores). These analyses were also performed using R (version 4.2.3) with the *lme4* (1.1–33) package.

## Data Availability

The data that support the findings of this study are available by request from the corresponding author.
